# Osteopathic Manipulative Medicine and Its Role in Psychiatry

**DOI:** 10.7759/cureus.47045

**Published:** 2023-10-14

**Authors:** Michael R Bowes, Mark R Speicher, Lan-Anh T Tran, Patcho N Santiago

**Affiliations:** 1 Behavioral Health, National Capital Consortium, Bethesda, USA; 2 Learning, Innovation, and Research, American Association of Colleges of Osteopathic Medicine, Bethesda, USA; 3 Behavioral Health, Uniformed Services University of the Health Sciences, Bethesda, USA

**Keywords:** adjunct treatments, complementary and alternative medicine (cam), psychiatry, psychosomatic medicine, osteopathic manipulation

## Abstract

This paper reviews the current literature to examine what elements of osteopathic medicine can be used in psychiatry. The aim of this study was to use the Preferred Reporting Items for Systematic Reviews and Meta-Analyses (PRISMA) guidelines to conduct a systematic review of studies describing the efficacy of osteopathic manipulative medicine (OMM) in treating psychiatric problems directly and indirectly. The authors searched the databases PubMed, PsycINFO, and CINAHL (Cumulative Index to Nursing and Allied Health Literature), reviewing peer-reviewed articles from 1980 to April 2023.

The literature demonstrates that OMM has a positive effect on psychiatric symptoms indirectly when treating certain medical conditions, such as chronic pain, fibromyalgia, and irritable bowel syndrome; however, there are many limitations on these studies, and further research is required prior to making firm recommendations. The evidence is lacking for osteopathic manual medicine being used directly to treat psychiatric conditions. This review demonstrates that in some populations, such as individuals with chronic pain, fibromyalgia, and irritable bowel syndrome, OMM could be considered by an osteopathic psychiatrist as an adjunct treatment. More research should be conducted in this area due to the many limitations in the available studies but current research suggests that the use of OMM by osteopathic psychiatrists could be beneficial for some patient populations.

## Introduction and background

For most of contemporary American medical history, psychiatrists were trained in medical schools leading to a Doctor of Medicine (M.D.) degree. Recently (since the latter part of the 20th century), however, this has changed and many psychiatrists in the United States are now osteopathically trained and receive a Doctor of Osteopathic Medicine (D.O.) degree, and this number is growing [1,2]. Despite curricular differences between M.D. (allopathic) and D.O. (osteopathic) medical schools, both graduates participate in the same residency programs under the Single Accreditation System (SAS) through the Accreditation Council for Graduate Medical Education (ACGME) [3]. This growth of osteopathic medicine’s presence in psychiatry training creates the question of whether anything from osteopathy should be used in psychiatry [4].

Osteopathy was founded in 1874 by Andrew Taylor Still who had an M.D. Still, he was a physician and surgeon, who served during the civil war [5]. He became disenchanted with the state of medical science at the time since many of the contemporary “cures” (opium, mercury, arsenic, etc.) and surgical procedures often resulted in more harm than benefit [6]. He developed a philosophy of medicine known as osteopathy, which involved manually working with the musculoskeletal system for diagnosis and treatment and allowing the body to heal itself. The core philosophy of osteopathy is outlined in the four osteopathic tenets [7]: (1) the body is a unit, and the person is a unit of body, mind, and spirit; (2) the body is capable of self-regulation, self-healing, and health maintenance; (3) structure and function are reciprocally interrelated; (4) rational treatment is based upon an understanding of the basic principles of body unity, self-regulation, and the interrelationship of structure and function.

Osteopathic physicians in the US use touch and hand placement as an integral part of their medical treatment of the patient. These techniques are known as osteopathic manipulative medicine (OMM) and include a wide array of hands-on techniques. In psychiatry, however, touch is usually discouraged [8]. This is because the relationship between a psychiatrist and a patient is unique, and there is concern about boundary violations. For osteopathic psychiatrists, this leads to the question of what elements of their unique osteopathic training should be incorporated into their psychiatric practice. Their osteopathic training emphasizes hands-on touch, while the psychiatric training discourages this. This paradox led us to explore whether there are situations in which hands-on OMM could be used by a psychiatrist to treat psychiatric disorders.

Research question and study aims

This study incorporates a review of previous research to determine whether OMM is effective for treating psychiatric disorders. The authors posit that osteopathic distinctiveness in psychiatry will improve patient outcomes through the use of OMM [9,10]. With these ideas in mind, the authors broadly searched PubMed, CINAHL (Cumulative Index to Nursing and Allied Health Literature), and PsycINFO. From the searches, the authors identified one major question: Can OMM treatment improve scores on measures of depression or anxiety? The authors divided this question into three broad categories covering OMM’s psychiatric effect as a treatment. With these categories, the authors then created the following research questions: (1) Can OMM cause positive outcomes in measures of depression or anxiety in healthy populations? (2) Can OMM indirectly cause positive outcomes in measures of depression or anxiety when treating other medical conditions? (3) Can OMM directly cause positive outcomes in measures of depression or anxiety when treating psychiatric conditions directly?

Materials and methods

Inclusion Criteria

The authors used the Preferred Reporting Items for Systematic Reviews and Meta-Analyses (PRISMA) guidelines throughout the creation of this paper [11]. The authors included studies that were published in peer-reviewed journals from 1980 to April 2023 that included open-label and randomized controlled trials. The searches were performed from December 2020 to April 2023, by the principal author. The principal author excluded the articles that did not meet inclusion criteria and all authors reviewed the remaining articles to determine which articles ultimately would be included. Disagreements between reviewers were settled by consensus. Different arguments were made and then voted on by the authors, ultimately all articles included in the review were accepted unanimously.

Search Strategy

Studies were identified by searching PubMed, PsycINFO, and CINAHL databases, using the following Boolean search terms: osteopathic, osteopathy, osteopathic manipulative medicine, OMM, osteopathic manipulative treatment, and osteopathic manipulative treatment associated with any of the following terms: psychiatry, psychiatric, fibromyalgia, psychogenic, psychosomatic, anxiety, depression, ADHD or attention deficit hyperactivity disorder, and schizophrenia.

Study Selection

**Figure 1:**
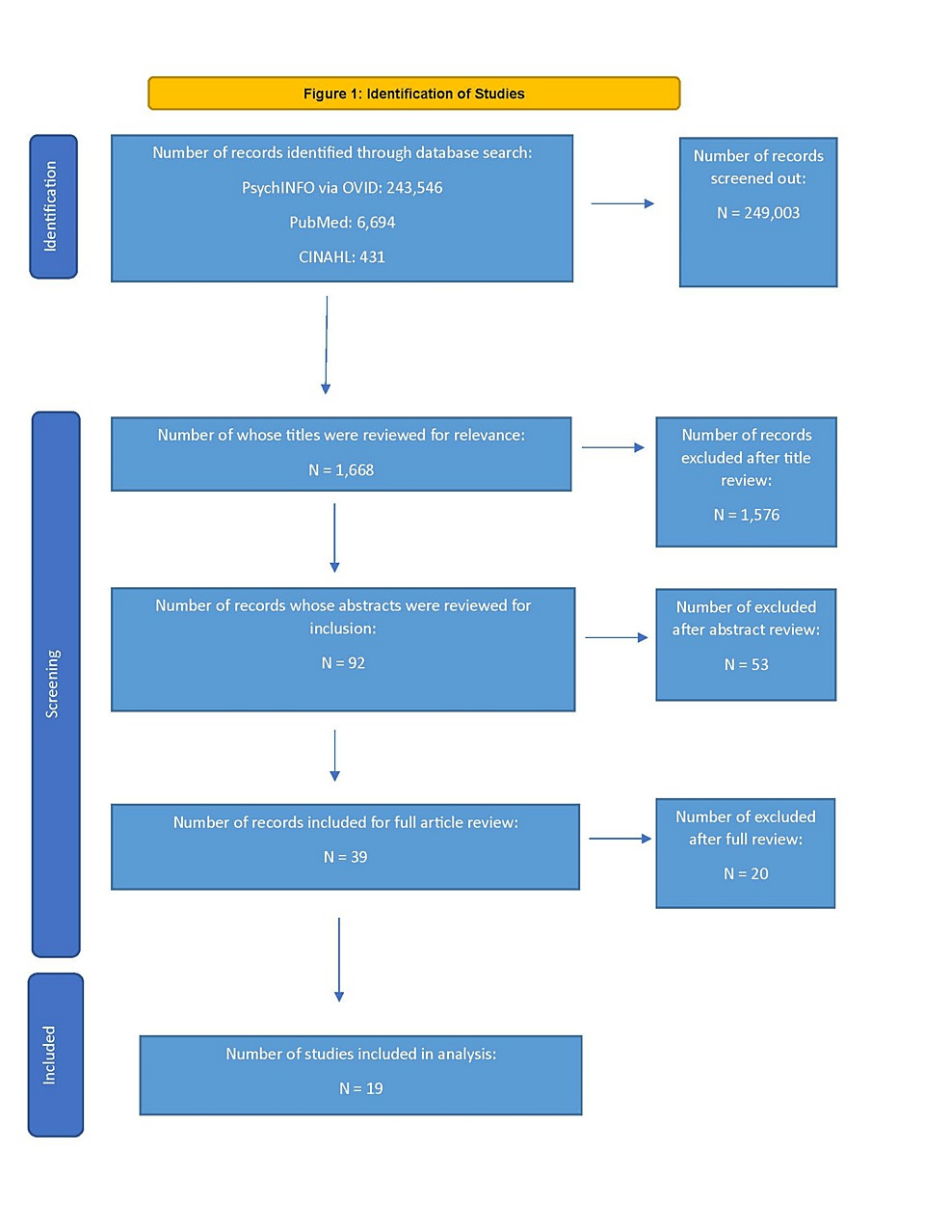
Identification of studies

Of the many results from the initial search (243,546 from PsycINFO, 6,694 from PubMed, and 431 from CINAHL), the authors selected only articles whose tested hypothesis(es) pertained to at least one of the three research questions. Of these, the authors found 92 abstracts and excluded 53 of them that did not meet the inclusion criteria. A full article review was conducted of the remaining 39 articles, which led to the exclusion of an additional 20 articles, leaving 19 articles in the final analyses. Reasons why these 20 articles were rejected included: studies that treated a neurologic problem rather than a psychiatric condition, studies assessing non-psychiatric treatment outcomes, interventions performed by non-osteopaths, and studies with very small sample sizes (e.g., N = 9 or less) (Figure 1).

## Review

Results

We found articles related to our three questions. Almost all of the articles in the literature search examined OMM's impact on anxiety and depression; we found no articles examining OMM’s impact on bipolar disorder, trauma-related disorders, or psychotic disorders. There were many different types of outcome measures used in the selected articles. These include physiological markers (e.g., heart rate) and overall well-being surveys, but most studies used psychiatric surveys as outcome measures. Every study in the review uses at least one specific OMM technique. Most studies do not specify a specific technique but rather use an osteopathic evaluation performed by an osteopathic provider who then performs indicated OMM techniques. Some studies compare OMM to sham OMM. Sham OMM is designed to mimic OMM; however, it is not an actual OMM technique.

Regarding the direct psychiatric effects of osteopathic manipulation, the authors found four studies [12-15] (Table 1). Different measures were used in each study and they include the Epworth Sleepiness Test, Patient Health Questionnaire-9 (PHQ-9), Self-Perceived Stress Scale, and physiologic markers (blood pressure, salivary alpha-amylase, and heart rate). Control groups consisted of either no intervention or having the subject be in a “relaxed position.” All these studies were performed on healthy test patients. Fernandez-Perez et al. [12] compared myofascial induction techniques to no intervention (sitting down) to determine differences in state anxiety (as measured by the State-Trait Anxiety Inventory), state depression (as measured by the Beck Depression Inventory (BDI)), systolic and diastolic blood pressure, and heart rate. There are statistically significant improvements seen in state anxiety, heart rate, and systolic blood pressure compared to the placebo group, but no significant difference in diastolic blood pressure, temperature, or state depression. Limitations of this study are that it is only one session, its sample size is only 41 participants, and the experiment is not blinded. Dugailly et al. [13] compared two randomly assigned groups of healthy females (an intervention group receiving OMM and a control group in a restful state) for a single session to evaluate differences in anxiety and global self-perception scores as measured by the Body Satisfaction and Global Self-Perception Questionnaire. The OMM group demonstrated a statistically significant improvement in anxiety and global self-perception scores, but no statistically significant difference is seen for body satisfaction. This study is limited by its small size (34 participants), duration of one session, and its unblinded design. Wiegand et al. [14] performed a randomized trial in which the authors compared OMM, non-directed OMM, and no treatment to evaluate improvement in sleep (Epworth Sleepiness Test), stress (Self-Perceived Stress Scale), and depression (PHQ-9). No differences were seen between the intervention groups and the control group for stress and depressive symptoms, but there was a statistically significant difference between the directed OMM group and the other two groups for sleep per the Epworth Sleepiness Test. The main limitations are the small size of the groups and the unblinded design. The last study by Abenavoli et al. [15] is an RCT consisting of three arms (OMM group receiving cranial osteopathic treatment, sham treatment group, and no intervention group) over one session. This study measured the salivary alpha-amylase, which the researchers use as a gauge for autonomic nervous system function. Both the sham treatment and the OMM groups experienced statistically significant increases in salivary alpha-amylase relative to the control group; however, there was no difference between the OMM and the sham groups. Like the previous studies, the limitations of this study include the duration of only one session and the use of an outcome measure that is not universally accepted.

**Table 1:** Osteopathic manipulation’s direct effect on mental state Ɨ Effect sizes calculated by the authors (where sufficient data were provided); other effect sizes in the original work. RCT: randomized controlled trial; OMM: osteopathic manipulative medicine; STAI: State-Trait Anxiety Inventory; BDI: Beck Depression Inventory.

Study (year)	Title	Journal	Study Type	Intervention	Duration	Size	Outcome Measures	Effect Size(s)	Findings
Fernandez-Perez et al. (2008) [12]	Effects of myofascial induction techniques on physiologic and psychologic parameters: a randomized controlled trial	Journal of Alternative Complementary Medicine	RCT	OMM vs. sitting in a relaxed position (control)	Single session	41 healthy males	Temperature, heart rate, systolic and diastolic blood pressure, State-Trait Anxiety Inventory (STAI), and the Beck Depression Inventory (BDI) measured before, during, and after interventions	Insufficient data presented	The OMM group had statistically significant improvements in state anxiety (seen in STAI), heart rate, and systolic blood pressure compared to the control group. No significant change was seen in diastolic blood pressure, temperature, and state depression as measured by BDI
Dugailly et al. (2014) [13]	Effect of a general osteopathic treatment on body satisfaction, global self-perception and anxiety: a randomized trial in asymptomatic female students	International Journal of Osteopathic Medicine	RCT	OMM vs. Restful State. The OMM techniques are described as "gentle repetitive mobilizations of the upper and lower extremities"	Single session	34	Self-questionnaires about anxiety, body satisfaction, and global self-perception	d_body satisfaction _= 0.55, d_self-perception_ = 1.25, d_anxiety_ = -1.07	Both groups had comparable improvements in body satisfaction, but the OMM group had a statistically significant larger effect on the measures of anxiety and global self-perception
Wiegand et al.​​​​​​​​​​​​​​ (2015) [14]	Osteopathic manipulative treatment for self-reported fatigue, stress, and depression in first-year osteopathic medical students	Journal of the American Osteopathic Association	RCT	Directed OMM, non-directed OMM, and no treatment. The directed OMM protocol focused on sympathetic techniques and cervical techniques	4 weeks	30	Epworth Sleepiness Test, Self-Perceived Stress Scale, and Primary Care Evaluation of Mental Disorders Patient Health Questionnaire 9 (which were administered before treatment, after two treatments, and after four treatments)	Insufficient data presented	The OMM group had statistically significant improvements in the Epworth Sleepiness Test compared to the placebo and the non-directed-OMM group, but no difference was seen on the Self-Perceived Stress Scale or Patient Health Questionnaire 9 tests for either OMM group compared to placebo
Abenavoli et al.​​​​​​​​​​​​​​ (2020) [15]	Cranial osteopathic treatment and stress-related effects on autonomic nervous system measured by salivary markers: a pilot study	Journal of Bodywork and Movement Therapies	RCT	OMM vs. sham treatment vs. control. The OMM technique used is called the CV4 technique	1 session	90	Salivary alpha-amylase (marker of autonomic nervous system function)	d_cv4_ = 2.64^Ɨ^	The sham and OMM groups experienced a statistically significant increase in salivary alpha-amylase compared to the control group, but there was no significant difference between the OMM and the sham therapy groups

Most of the published papers focus on osteopathic manipulation’s effect on psychiatric symptoms when treating comorbid medical conditions (Table 2). A major limitation of these studies is that the psychiatric outcomes are all secondary outcome measures. Eight randomized controlled trials (RCTs), two open-label studies, and one pre-post study met the inclusion criteria [16-26]. In six of the 11 studies, the group receiving OMM demonstrated a statistically significant improvement in the psychiatric outcomes relative to the control group. The medical conditions treated in these interventions were fibromyalgia, chronic pain, multiple sclerosis (MS), headaches, whiplash injury, and irritable bowel syndrome (IBS).

**Table 2:** Osteopathic manipulation’s indirect effect on psychiatric conditions when treating other medical problems Ɨ Effect sizes calculated by the authors (where sufficient data were provided); other effect sizes in the original work. RCT: randomized controlled trial; OMM: osteopathic manipulative medicine; SF-12: 12-Item Short Form Survey; SF-36: Short Form Health Survey. dEDSS: Extended Disability Status Score; dMFIS: The Modified Fatigue Impact Scale; dBDI: The Beck Depression Inventory-II; dBAI: The Beck Anxiety Inventory ("d" in each abbreviation denotes that the resulting effect size is a Cohen's d).

Study	Title	Journal	Study Type	Intervention	Duration	Size	Outcome Measures	Effect Size(s)	Findings
Gamber et al. (2002) [16]	Osteopathic manipulative treatment in conjunction with medication relieves pain associated with fibromyalgia syndrome: results of a randomized clinical pilot project	The Journal of the American Osteopathic Association	RCT	The first intervention group received OMM. The second intervention group received OMM and teaching. The third group received moist heat, and the fourth group was a control group that received treatment as usual	23 Weeks	24	Depression was assessed using the Center for Epidemiological Studies Depression Scale (CESDS)	Insufficient data presented	There was not a statistically significant difference between the groups in terms of depression scores
Williams et al. (2003) [17]	Randomized osteopathic manipulation study (ROMANS): pragmatic trial for spinal pain in primary care	Family practice	RCT	The intervention group received OMM and the control group received treatment as usual. The OMM was primarily "spinal manipulation"	6 months	201	SF-12 mental score	d_SM-12mental 6 mo _= 0.42 ^Ɨ^, d_SMPQtotal 6 mo _= 0.31^ Ɨ^	OMM done in 3 sessions statistically significantly improved SF-12 scores relative to the treatment as usual group
Florance et al. (2012) [18]	Osteopathic treatment improves the severity of irritable bowel syndrome: a pilot randomized sham-controlled study	European Journal of Gastroenterology and Hepatology	RCT	The intervention group received OMM and the control group received sham OMM. The OMM techniques were direct and indirect spinal maneuvers as well as visceral osteopathic maneuvers	28 days	30	Patient self-measures for anxiety and depression	d_IBS severity _= 1.70^ Ɨ^, d_quality of life _= 0.28^ Ɨ^	Depression and anxiety scores decreased with the sham OMM and OMM groups; however, there was no statistical difference between the two groups
Schwerla et al. (2013) [19]	Osteopathic treatment of patients with long-term sequelae of whiplash injury: effect on neck pain disability and quality of life	Journal of Alternative and Complementary Medicine	Pre-post study	The intervention group received OMM and the control group received no treatment	6 weeks for each stage	42	The mental health portions of the SF-36	d_NPAD_ = -0.78^ Ɨ^, d_SF-36 Physical _= 0.30^ Ɨ^, d_SF-36 Mental _= 0.60^ Ɨ^	Per the SF-36 mental component, there was a statistically significant improvement after the OMM portion of the treatment
Moustafa et al. (2015) [20]	The addition of upper cervical manipulative therapy in the treatment of patients with fibromyalgia: a randomized controlled trial	Rheumatological International	RCT	OMM + multimodal program vs. control (multimodal program). The OMM group received upper cervical manipulative therapy interventions	1 year	120	Beck Anxiety Inventory, Beck Depression Inventory, Pittsburgh Sleep Quality Index	d_FIQ_ = 0.95, d_PCS_ = 0.93, d_Algorithmic score _= -0.70	The OMM group had statistically significant improvement after 12 months for the measures of anxiety, depression, and sleep
Espi-Lopez et al.​​​​​​​ (2016) [21]	Efficacy of manual therapy on anxiety and depression in patients with tension-type headache. A randomized controlled clinical trial	International Journal of Osteopathic Medicine	RCT	3 groups of OMM (articulatory techniques, soft tissue techniques, and a combination of the two) and a control group. In this study, 2 different cranial OMM techniques are used in the intervention group (suboccipital release and occiput-atlas-axis joint manipulation)	4 weeks	84	State-Trait Anxiety Inventory and Beck Depression Inventory	Sensory dimension: d_manual therapy _= 0.59, d_manipulative therapy _= 0.75, d_combination_ = 0.45, d_control_ = 0.62. Affective dimension: d_manual therapy _= 0.24, d_manipulative therapy _= 0.77, d_combination_ = 0.60, d_control_ = 0.18. Evaluative dimension: d_manual therapy _= 0.91, d_manipulative therapy _= 1.00, d_combination_ = 0.71, d_control_ = 0.33. Number of word descriptors: d_manual therapy _= 0.66, d_manipulative therapy _= 0.89, d_combination_ = 0.58, d_control_ = 0.47. Intensity of pain: d_manual therapy _= 0.86, d_manipulative therapy _= 0.87, d_combination_ = 1.60, d_control_ = 0.61	All OMM groups and the control group caused moderate improvements in depression and anxiety among headache sufferers. There were no significant differences seen between the different osteopathic techniques or with the control
Cordano et al.​​​​​​​ (2018) [22]	Osteopathic manipulative therapy and multiple sclerosis: a proof-of-concept study	The Journal of the American Osteopathic Association	Open-label study	The intervention group received OMM and the control group received multiple sclerosis education. The OMM techniques were "passive techniques" of the upper and lower limbs	6 months	21	The Beck Depression Inventory-II, the Beck Anxiety Inventory, and the SF-12	dEDSS =-0.06Ɨ dMFIS = 0.27Ɨ dBDI = -0.09Ɨ dBAI = 0.62Ɨ	The OMM group experienced statistically superior effects in fatigue and depression compared to the control group. No statistical significance was found with anxiety.
Marti-Salvador et al.​​​​​​​ (2018) [23]	Osteopathic manipulative treatment including specific diaphragm techniques improves pain and disability in chronic nonspecific low back pain: a randomized trial	Archives of Physical Medicine and Rehabilitation	RCT	The intervention group received OMM with diaphragm technique and the control group received sham OMM. The OMM group received diaphragmatic techniques and techniques targeting the lower back	4 weeks	66	Hospital Anxiety and Depression Scale	Insufficient data presented	Significant reductions in the OMM group compared to the sham group for both anxiety and depression scores
Porcari et al.​​​​​​​ (2019) [24]	Effects of osteopathic manipulative treatment on patients with multiple sclerosis: a pilot study	Complementary Therapies in Medicine	Open-label prospective study	The control group received 5 days a week of conventional rehabilitation, while the experimental group received 3 days a week of conventional rehabilitation and 2 days of specific OMM	8 weeks	20	Hamilton anxiety rating scale	Insufficient data presented	Statistically significant reductions were noted in the Hamilton anxiety rating scale scales for the OMM group compared to the control group
Chvetzoff et al.​​​​​​​ (2019) [25]	Osteopathy for chronic pain after breast cancer surgery: a monocentric randomized study	Bulletin du Cancer	RCT	2 groups of patients following breast surgery. One received standard analgesic treatment and the other arm received OMM as well	12 months	28	Hospital anxiety and depression scale (HADS)	Insufficient data presented	No change in pain scores between the groups, but there was a statistically significant improvement in depression scores seen in the OMM treatment group
Cholewicki et al. (2022) [26]	The effects of osteopathic manipulative treatment on pain and disability in patients with chronic neck pain: a single-blinded randomized controlled trial	PM & R: The Journal of Injury, Function, and Rehabilitation	Single-blinded, cross-over, randomized-controlled trial	The first arm received OMM and the second group (control group) was placed on a waiting period list. After 4-6 weeks, the patient groups switched	4-6 weeks	75	The anxiety and depression portions of the Patient-Reported Outcomes Measurement Information System-29 (PROMIS-29)	d_average pain _= 0.67, d_current pain _= 0.65, d_NDI% _= 0.62, d_PROMIS-sleepdistrurbance _= 0.72, d_PROMIS-fatigue _= 0.55	The intervention OMM group had a statistically significant improvement in the depression and anxiety portions of the PROMIS-29 questionnaire compared to the control group

Gamber et al. [16] compared four groups of fibromyalgia patients, two of whom received OMM. After 23 weeks of treatment, there was no statistically significant difference between the groups in depression scores (measured by the Center for Epidemiological Studies Depression Scale). The duration was a strength, but the groups' sizes were a weakness (six participants in each subject group). One of the stronger studies in the review is by Williams et al. [17], who evaluated OMM with spinal pain patients. The study randomizes 201 patients to either a treatment-as-usual group or an OMM group receiving cranial osteopathic treatment. The patients were followed for six months. The scale that the investigators used was the Short Form-12, which is a 12-question survey with eight different domains, and one of those domains is "general mental health" (such as psychological distress and well-being). Investigators found a statistically significant improvement in general mental health symptoms for the participants who received OMM. The main limitation of this study is the seldom-used outcome measure in psychiatry and thus it does not have the validity of other common psychiatric scales. Florance et al. [18] compared OMM and sham OMM head-to-head in 30 IBS patients to evaluate changes in IBS symptoms, as well as depression and anxiety self-reports as secondary measures. Patients in both groups experienced an improvement in anxiety and depression scores, which were not statistically significantly different. A more standardized metric would have improved the validity of this study. Schwerla et al. [19] performed a quasi-experimental pre-post study of 42 patients with whiplash injury. The psychiatric outcome measure was the mental health domain of the Short Form 36 (SF-36), and statistically significant improvement was demonstrated. Limitations were the study design (not randomized or blinded) and the outcome measure (a subset of the general SF-36 scale, and not a more validated psychiatric symptom measure). Moustafa et al. [20] randomized 120 fibromyalgia patients into two groups, which both received a typical multimodal program, but the intervention group also received cervical OMM. The investigators found no statistically significant differences in the psychiatric measures after 12 weeks (Beck Anxiety Inventory, BDI, Pittsburgh Sleep Quality Index) but after one year, there was a statistically significant improvement in each category. This study is well-designed and well-powered but includes the psychiatric scales as secondary measures. Espi-Lopez et al. [21] conducted a four-week RCT on tension headache sufferers. Patients were randomized into one of four groups (three of the groups used a specific OMM technique, while the fourth group was a control group). This study uses well-validated scales to evaluate anxiety (State-Trait Anxiety Inventory (STAI-SA and STAI-TA)) and depression (BDI). The study lasted four weeks and included multiple sessions. Despite some improvement in anxiety and depression scores in all groups, there is no statistically significant difference in the improvement between the osteopathic groups and the controls. Cordano et al. [22] performed an open-label study for MS patients. In the study, one group received OMM while the other group received MS education. The OMM group experienced statistically significant improvements in depression, fatigue, and quality of life scores compared to the education group. The limitations are that this is a proof-of-concept study and thus was not randomized. Marti-Salvador et al. [23] evaluated diaphragmatic osteopathic techniques for lower back pain. The investigators randomized 66 chronic low back pain patients into two groups with one receiving actual OMM (diaphragmatic techniques) and the other receiving sham OMM. Significant improvement are demonstrated in anxiety and depression scores (Hospital Anxiety and Depression Scale (HADS)) among the OMM group. The duration of four weeks and the group sizes are relative strengths. Porcari et al. [24] designed an open-label prospective study comparing two different rehabilitation protocols in MS patients; both groups received five days of rehabilitation, but the intervention group also received OMM in two of those days. The OMM group demonstrated a statistically significant reduction in the Hamilton Anxiety Rating Scale. The open-label design of the study is a key limitation. Chvetzoff et al. [25] performed an RCT on 28 patients with chronic pain following breast cancer surgery. Groups were randomized to a treatment-as-usual group and a group that also received OMM. Investigators found no difference in pain scores; however, the OMM group experienced a statistically significant improvement in depression scores. This study is limited by its size of only 28 patients, but it did have an adequate duration of 12 months. Cholewicki et al. [26] performed a single-blind cross-over RCT of 75 chronic neck pain patients. One arm received OMM and the patients in the other were placed on a waiting list. The OMM intervention group had a statistically significant improvement in the anxiety and depression scales of the PROMIS-29 questionnaire (a survey that assesses pain intensity as well as seven other health domains, two of which are depressive and anxiety symptoms). Limitations of this study were that there was no active control, and the outcome measure was only a subset of a general questionnaire.

There are limited data regarding OMM’s direct effects on psychiatric conditions (Table 3), and only four studies met our inclusion criteria [27-30]. One study assessed children with attention-deficit/hyperactivity disorder (ADHD), another assessed women with depression, and two examined adults with anxiety. Plotkin et al. [27] compared depression response in two groups of patients over eight weeks; one group (eight patients) received OMM, psychotherapy, and Paxil while the second group (nine patients) received Paxil and psychotherapy without OMM. The OMM group demonstrated a statistically significant decrease in Zung Self-Rating Depression Scale (SDS) scores compared to the control group. Strengths of this study are that the patients were blinded to treatment status and the outcome measure is well validated. Limitations of the study include the size (17) and the high dropout rate. Accorsi et al. [28] evaluated 28 children with ADHD. One group received "conventional care" while the other received "conventional care" + OMM. The researchers found that OMM did not improve the primary outcome among ADHD patients (accuracy and rapidity on the Biancardi-Stropa test) on univariate analysis; however, statistically significant improvement in patients receiving OMM was shown in multivariate linear regression. A key limitation of this study (in addition to its size) is the measure used, the Biancardi-Stroppa Modified Bell Cancellation Test. The Biancardi-Stroppa Modified Bell Cancellation Test has not been validated outside Italy and its validity in other populations is unknown. Dixon et al. [29] and Gozalo-Pascual et al. [30] examined whether OMM could benefit patients with clinical anxiety (generalized anxiety disorder in Dixon's study and "clinical anxiety” in Gozalo-Pascual's study). Dixon et al. undertook an open-label, non-randomized trial that provided five OMM sessions to 26 patients who met the criteria for generalized anxiety disorder. These patients had a clinically significant reduction in anxiety symptoms; however, the study had no control group, was not blinded, and had a small sample size. Gozao-Pascual et al. performed an RCT in which the intervention group received four OMM sessions (myofascial release), and the control group received a sham treatment. The OMM group experienced a substantial reduction in anxiety compared to the control group. This was a randomized trial that provided active control; however, the study was not blinded and again suffered from a small group of patients (36).

**Table 3:** Osteopathic manipulation’s direct effects on psychiatric conditions Ɨ Effect sizes calculated by the authors (where sufficient data are provided); other effect sizes in the original work. RCT: randomized controlled trial; OMM: osteopathic manipulative medicine; STAI: State-Trait Anxiety Inventory; HAM-A: Hamilton Anxiety Rating Scale.

Study (year)	Title	Journal	Study type	Intervention	Duration	Size	Outcome measures	Effect size(s)	Findings
Plotkin et al. (2001) [27]	Adjunctive osteopathic manipulative treatment in women with depression: a pilot study	The Journal of the American Osteopathic Association	RCT	Two groups and both groups received medication (Paxil) and therapy (control group) but the intervention group also received OMM. No specific OMM protocol was performed	8 weeks	17	The Zung Depression Scale	Insufficient data presented	After eight weeks of treatment, there was a statistically significant improvement in the Zung Depression Scale scores among the treatment group compared to the control group
Accorsi et al.​​​​​​​ (2014) [28]	Effect of osteopathic manipulative therapy in the attentive performance of children with attention-deficit/hyperactivity disorder	The Journal of the American Osteopathic Association	RCT	2 groups of children ages 5-15 were randomly assigned either to an OMM + conventional care or to a conventional care-only group. No specific OMM protocol was used	10 weeks	28	Biancardi-Stroppa Modified Bell Cancellation Test accuracy and rapidity scores	Insufficient data presented	No differences were seen on univariate analysis between the two groups, but multivariate testing showed that the OMM group had statistically significant improvements in Biancardi-Stropa test accuracy, rapidity, and points
Dixon et al.​​​​​​​ (2020) [29]	Effect of osteopathic manipulative therapy on generalized anxiety disorder	Journal of Osteopathic Medicine	Open-label, nonrandomized black-box study	Subjects were screened by the Hamilton Anxiety Rating Scale (HAM-A), and if they met the criteria for generalized anxiety disorder, they then received 5 individual OMM sessions. No specific OMM protocol was used	8-9 weeks	26	Hamilton Anxiety Rating Scale (HAM-A) Beck Anxiety Inventory (BAI)	Insufficient data presented	Patients who underwent the 5-6 sessions of OMM had a "significant reduction" in anxiety symptoms per the HAM-A and BAI inventories
Gozalo-Pascual et al. (2023) [30]	Efficacy of the myofascial approach as a manual therapy technique in patients with clinical anxiety: a randomized controlled clinical trial	Complementary Therapies in Clinical Practice	Randomized placebo-controlled clinical trial	The treatment consisted of four myofascial sessions (OMM) of 40 minutes for four weeks. The placebo group received four sessions of simulated myofascial intervention (sham treatment) of the same duration and frequency as the treatment group	6 weeks	36	STAI	d_STAI-state anxiety _= 0.76, d_VAS_ = 1.94	The OMM group experienced a clinically significant reduction in anxiety compared to the placebo group

Discussion

This review has multiple limitations. These include publication bias, a low degree of rigor in many of the studies, small sample sizes, and a wide variation of outcome measures.

The authors found articles relating to all three of our research questions, but due to the limitations identified above, none of the questions could be answered by this research. There currently is insufficient data to make any recommendation favoring OMM’s role in the direct treatment of psychiatric symptoms. Four articles demonstrate that osteopathic manipulation can improve psychiatric symptoms in the general population. Despite the positive results, these studies have major limitations such as non-uniform outcome measures, which include differing scales of varying quality and physical measures (such as heart rate and blood pressure) that are not widely accepted as validated endpoints. Additionally, most of these studies were limited by their size. The literature was sparser regarding whether OMM improves psychiatric disorders directly. We found only four studies of relatively low quality, but all demonstrated that OMM can improve anxiety, depression, and ADHD symptoms.

The largest body of literature investigated the indirect effect of OMM on psychiatric symptoms when co-morbid medical conditions are being treated. Eleven studies are included in this review (eight RCTs). Most of the studies (six) demonstrate statistically significant improvement in psychiatric symptoms when the primary medical condition is treated with OMM, whether or not the primary condition improves. While using OMM directly to treat psychiatric conditions requires much more study to make any conclusions, there appears to be some literature supporting osteopathic psychiatrists using OMM medicine with patients who have other medical conditions.

OMM includes many different techniques in different parts of the body. Throughout the review, many techniques are discussed, but most commonly the OMM provided is based on the OMM practitioner's clinical judgment (no standardized treatment). Some commonly seen treatments in the review include different cranial treatments, spinal OMM, and diaphragmatic techniques. Perhaps these could be a focus of future OMM psychiatric research.

Touching patients is essential to the use of OMM; however, it is oftentimes discouraged in psychiatry [8]. Touch is avoided in psychiatry because of potential boundary issues, and the relationship between a patient and their psychiatrist is different from the relationship between patients with other physicians. As a result, many psychiatrists do not use OMM in their practice [31]. The literature in this review suggests, however, that in some patient populations (such as those with chronic pain and psychosomatic disorders), osteopathic manipulation could be used by an osteopathic psychiatrist. If the psychiatrist chooses not to directly perform the OMM, then an OMM consult with another osteopathic physician could provide the same level of benefit while preserving the traditional boundaries between a psychiatrist and their patient.

## Conclusions

Osteopathic psychiatrists learn how to perform OMM during their medical school training; however, few osteopathic psychiatrists utilize OMM in their practice. To our knowledge, no systematic review has examined the use of OMM in psychiatric disorders. We conducted this review to determine if OMM can improve psychiatric symptoms, and with what patient populations. This review demonstrates that OMM utilized by osteopathic physicians could improve psychiatric symptoms indirectly if other comorbid conditions (i.e., fibromyalgia, multiple sclerosis, chronic pain, or a psychosomatic illness) are treated with OMM. There was only weak evidence supporting the use of OMM to directly treat psychiatric conditions. There is also minimal evidence suggesting that OMM could be used to help patients achieve a more restful state. Prior to recommending OMM as a routine part of psychiatric treatment, more research will be needed, especially blinded RCTs with an adequate number of study subjects.
